# Bilateral Epicardial Coronary Microfistulas With Anomalous Deviation

**DOI:** 10.1016/j.jaccas.2025.106798

**Published:** 2026-01-29

**Authors:** João Cleriston da Silva Calheiros, João Pedro Kern Alves, Natan Lucca Lima, Darlos Kelvin de Azevedo, Luís Cláudio Izidio Costa Júnior, Bruna Magalhães Magota, Marina Germano Gomes, Gabriel Pettenon Gubert, Sofia Zilli Santos, Murilo Zomer Frasson

**Affiliations:** a Department of Medical Sciences, Federal University of Santa Catarina, Araranguá, Santa Catarina, Brazil; b University of the Extreme South of Santa Catarina, Criciúma, Santa Catarina, Brazil; c Department of Cardiology, São José Hospital, Criciúma, Santa Catarina, Brazil; d Emergency Department, São João Batista Hospital, Criciúma, Santa Catarina, Brazil; e Faculty of Medicine, Federal University of Santa Catarina, Araranguá, Santa Catarina, Brazil

**Keywords:** case report, coronary artery fistula, coronary vessel anomalies, microfistulas

## Abstract

**Background:**

Bilateral coronary microfistulas with redirection to the left ventricle are extremely rare clinical events. Although most cases are asymptomatic, the severity varies according to the redirection of blood flow, possibly leading to type 2 myocardial infarction.

**Case Summary:**

A 79-year-old woman with a medical history of hypertension, type 2 diabetes mellitus, hypothyroidism, and anxiety presented with atypical chest pain. Electrocardiogram revealed sinus rhythm with an electrically inactive area in the inferior wall, and serial troponin measurements indicated myocardial injury. Coronary angiography demonstrated bilateral coronary microfistulas draining into the left ventricle, in the absence of obstructive coronary artery disease. The patient was discharged with atenolol and acetylsalicylic acid and remained stable during follow-up.

**Discussion:**

This case contributes to the growing recognition of coronary microfistulas as a rare but clinically meaningful cause of type 2 myocardial infarction, emphasizing the diagnostic value of coronary angiography and reinforcing the importance of individualized conservative management in such diffuse presentations of fistulas.

## Visual Summary

**Figure:**
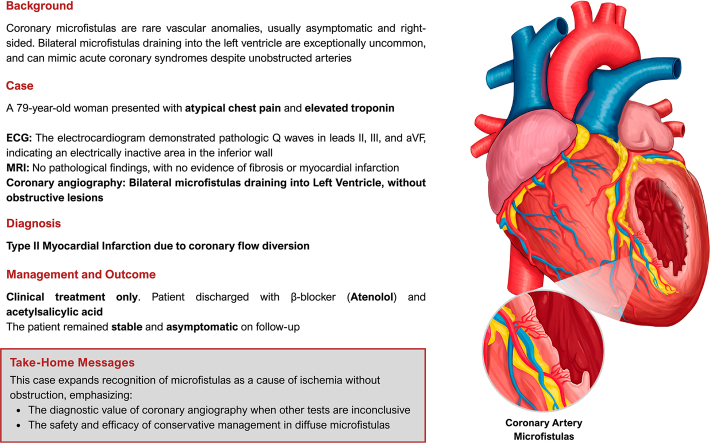

Coronary microfistulas are abnormal communications that create a hemodynamically mandatory shunt, diverting blood flow from a coronary artery toward a cardiac chamber or the great vessels.^1^ This finding may lead to myocardial ischemia without obstructions, that is, a type 2 myocardial infarction (MI). The severity of symptoms varies according to the amount of diverted blood flow, with 75% of cases being asymptomatic.^2^ Drainage into the right heart chamber is the most common form, whereas its occurrence in the left ventricle is rare.^3^ Its incidence is low in the population, with bilateral and multilateral forms being uncommon.^4^

**Visual Summary:**
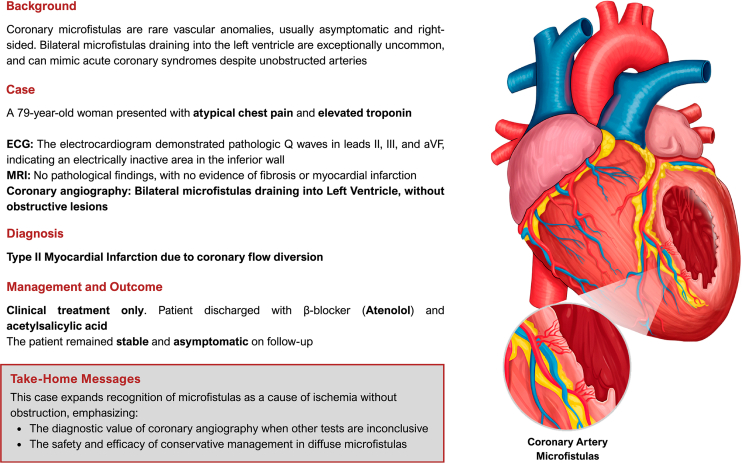
Coronary Artery Microfistulas With Left Ventricular Drainage ECG = electrocardiogram; MRI = magnetic resonance imaging.

## History of Presentation

A 79-year-old woman sought cardiology evaluation of atypical chest pain that had lasted 1 day. Cardiac magnetic resonance imaging (MRI) was requested. One day after undergoing the examination, she presented to the emergency department reporting continuous, burning precordial pain that began after the procedure.

## Past Medical History

The patient's medical history included hypertension, type 2 diabetes mellitus, hypothyroidism, and anxiety disorder. She was a former smoker (quit 40 years ago), and she reported a family history of death in a second-degree relative due to atherosclerotic coronary artery disease.

## Investigations

The initial physical examination revealed no significant abnormalities. The electrocardiogram (ECG) performed in the emergency department showed sinus rhythm with an electrically inactive area in the inferior wall, with pathological Q waves initially observed in leads II, III, and aVF. Serial high-sensitivity cardiac troponin I measurements demonstrated a progressive increase in levels, with values of 664 and 1,024 ng/L (reference range: <14 ng/L).

**Figure 1:**
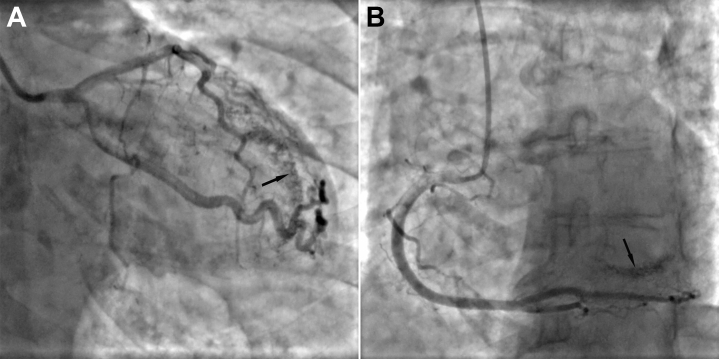
Coronary Angiography (A) Microfistulas visualized in the left coronary artery (arrow). (B) Microfistulas visualized in the right coronary artery (arrow).

Simultaneously, the cardiac MRI report was obtained, which showed no evidence of myocardial fibrosis or MI (Video 1). Considering the clinical presentation, the patient was admitted and underwent coronary angiography, which revealed bilateral high-flow microfistulous connections originating from both the left and right epicardial coronary systems and draining into the left ventricle, with no evidence of epicardial stenosis, delayed distal opacification, or slow-flow phenomena that would suggest primary microvascular disease—findings that were consistent with the diagnosis of type 2 MI (Figure 1, Video 2, Video 3, Video 4, Video 5).

Transthoracic echocardiography demonstrated grade 1 diastolic dysfunction, mild left atrial enlargement, hypokinesia of the inferior wall of the left ventricle, and a small pericardial effusion.

## Differential Diagnosis

The electrocardiographic findings, combined with the rise in troponin levels on serial measurements, raised the differential diagnosis of non–ST-segment elevation acute coronary syndrome or ST-segment elevation MI involving the inferior wall with delayed presentation. However, these hypotheses were later ruled out, as the MRI did not reveal any findings consistent with MI.

## Management

Three days after admission, the patient was asymptomatic and stable. Upon discharge, atenolol 50 mg and acetylsalicylic acid 100 mg were prescribed, in addition to the patient's prior medications: levothyroxine 100 μg, rosuvastatin 5 mg, trimetazidine dihydrochloride 35 mg, and empagliflozin + linagliptin 25/5 mg. She was also advised to schedule a follow-up appointment with her attending cardiologist. We decided to proceed with clinical management of the patient, aiming to monitor the progression of symptoms through regular follow-up with her specialist physician.

## Follow-Up

**Figure 2:**
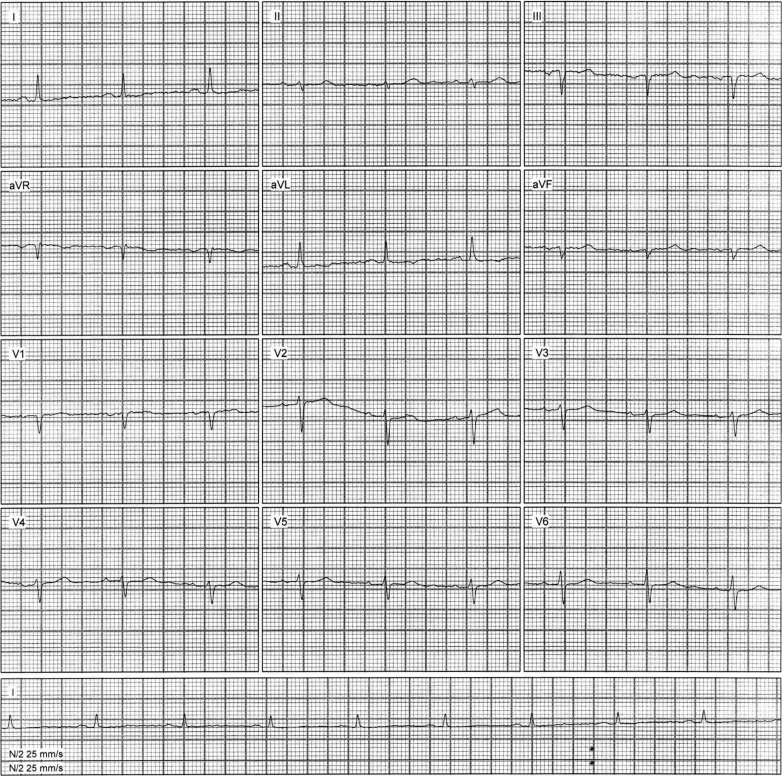
Follow-Up Electrocardiogram The electrocardiographic tracing demonstrates the absence of previously observed pathologic Q waves in leads II, III, and aVF, which had indicated an electrically inactive area in the inferior wall, suggestive of a prior myocardial infarction in this region.

During the follow-up consultation with the cardiologist, held 5 days after discharge, it was confirmed that the patient remained stable. A follow-up ECG performed during the outpatient evaluation showed normalization of the inferior Q waves previously described at admission, with no new repolarization abnormalities (Figure 2).

## Discussion

This case highlights a rare presentation of type 2 MI caused by bilateral coronary microfistulas draining into the left ventricle, underscoring the importance of considering vascular anomalies in the differential diagnosis of ischemia without obstructive coronary disease. Consistent with previous reports in the literature,^3^^,^^5^^,^^6^ this case illustrates how microfistulas can mimic ischemic syndromes, often presenting with chest pain. In addition to chest pain, microfistulas are often associated with other characteristic symptoms of MI, including chest tightness, palpitations, dyspnea, and dizziness, although clinical examination typically reveals no specific signs indicative of fistulas.^3^^,^^6^ Furthermore, coronary microfistulas may appear with other presentations, such as congestive heart failure, endocarditis, cardiac arrhythmia, pulmonary hypertension, and syncope.^6^^,^^7^

Coronary angiography, the gold standard for diagnosis, confirmed the fistulas in this case, correlating with the findings described by Chen et al,^3^ who reported no evident abnormalities in the course of the coronary arteries during catheterization but identified a distal microvascular fistula with contrast draining into the left ventricle during diastole—a phenomenon consistent with what was observed on our patient's angiographic examination. This hemodynamic alteration can lead to an imbalance between oxygen supply and demand, supporting the diagnosis of a type 2 MI. To further support this diagnostic impression, our patient's clinical findings were also consistent with those observed in cases of congenital coronary artery–left ventricular multiple microfistulas (CA-LVMMFs), as described by Said and van der Werf.^6^ In this context, angina pectoris—reported by our patient as a burning precordial pain—stands out as one of the most frequent symptoms, present in approximately 70% of CA-LVMMF cases.^6^ Additionally, electrocardiographic changes, such as the electrically inactive areas seen on our patient's ECG, are also common, occurring in 75% of the patients in the Said and van der Werf study. The echocardiographic findings revealed structural changes that are nonspecific and do not suggest a defined etiology on their own. However, considering the clinical and angiographic context, it is possible that these findings are secondary to the presence of coronary microfistulas draining into the left ventricle.^8^ Although Doppler echocardiography was not performed in the current case, the literature highlights its potential for identifying abnormal flow from the coronary arteries into the left ventricle, which could support the diagnosis of the observed condition.^3^^,^^5^

Percutaneous closure is commonly employed for treating large or symptomatic coronary artery fistulas. However, more invasive interventions such as surgery or transcatheter occlusion are generally not feasible for coronary multiple microfistulas given their complex anatomy and diffuse distribution. In most cases, symptom management for microfistulas is more effectively achieved through conservative medical therapy.^3^^,^^6^^,^^7^ The management chosen for our patient, based on clinical treatment and outpatient follow-up, is consistent with the recommendations found in the literature for cases of CA-LVMMFs. Previous reports have shown that the use of beta-blockers and calcium-channel blockers is effective in alleviating CA-LVMMF–related symptoms and may contribute to improved patient prognosis.^3^^,^^6^^,^^9^ Therefore, conservative pharmacological treatment is considered the first-line approach for this condition.

In the current case, the prescription of atenolol, a beta-blocker, aligns directly with this therapeutic strategy, aiming to reduce myocardial oxygen demand and prevent further ischemic events, within a plan of continuous clinical monitoring supported by cardiology follow-up. The choice of atenolol was further supported by the patient's clinical stability, absence of tachyarrhythmias, and the lack of angiographic or cardiac MRI findings suggestive of vasospasm or significant microvascular dysfunction—factors that reduce the rationale for initiating nondihydropyridine calcium-channel blockers. Moreover, in elderly patients with multiple comorbidities, nondihydropyridine calcium-channel blockers pose higher risks of bradycardia, atrioventricular block, and drug-drug interactions, making a beta-blocker a safer and more targeted option to address the oxygen supply-demand imbalance characteristic of microfistula-related ischemia. When these medications are contraindicated, alternatives such as ivabradine^10^ and ranolazine^7^ may be considered. Nitrates, and possibly similar vasodilator agents, should generally be avoided, as they may exacerbate symptoms by increasing coronary flow diversion.^7^ Other novel therapies, including enhanced external counterpulsation, low-intensity interval exercise, and targeted endothelial treatments, were not pursued, as the patient remained clinically stable and asymptomatic after medical optimization with atenolol, which effectively reduced myocardial workload and oxygen consumption. Low-dose acetylsalicylic acid was also initiated based on a preventive and pathophysiological rationale, as the abnormal vascular connections and turbulent flow associated with coronary-ventricular microfistulas may promote endothelial dysfunction and platelet activation, increasing the risk of microthrombosis or distal embolization. This approach is consistent with a previous report that empirically employed long-term antiplatelet use in a similar setting.^6^

Future studies should explore the prevalence and prognostic implications of microfistulas, mirroring calls for standardized protocols in fistula management. Together, these cases illustrate the spectrum of nonobstructive coronary pathologies necessitating individualized diagnostic and therapeutic strategies.

## Conclusions

This report aims to illustrate the complexity of type 2 MI associated with bilateral coronary microfistulas with drainage to the left ventricle, as well as to reinforce the importance of considering vascular anomalies in the diagnosis of type 2 MI. It is noted that the patient presented significant elevation of troponin and nonspecific electrocardiographic and echocardiographic alterations, but no confirmation of coronary obstruction or anomalies on cardiac MRI. When performing coronary angiography, the gold standard for diagnosing fistulas, the alteration was shown to be present, allowing the diagnosis of this rare condition as the etiology of the metabolic imbalance between oxygen supply and demand. Clinical management included clinical monitoring, control of risk factors, and drug treatment, which allowed a stable discharge and referral for specialized outpatient follow-up.

## Funding Support and Author Disclosures

The authors have reported that they have no relationships relevant to the contents of this paper to disclose.

**Take-Home Messages**

• Microfistulas, though rare, should be considered in type 2 myocardial infarction, particularly when conventional causes are absent. Imaging remains essential for definitive diagnosis.
• Conservative medical management is usually sufficient and effective for most cases.

## Appendix

Video 1 Cardiac magnetic resonance imaging revealing no pathological findings, with no evidence of fibrosis or myocardial infarction.

Video 2 Posteroanterior view angiography of the left coronary artery showing microfistulas. Contrast opacification reveals fine vascular channels draining into the ventricular cavity.

Video 3 Left anterior oblique projection angiography of the left coronary artery showing no signs of obstruction.

Video 4 Posteroanterior cranial view angiography of the left coronary artery showing microfistulas.

Video 5 Left anterior oblique caudal projection angiography of the right coronary artery showing microfistulas and no signs of obstruction.
